# Spatial knowledge and flood preparedness in Victoria Island, Lagos, Nigeria

**DOI:** 10.4102/jamba.v13i1.825

**Published:** 2021-03-05

**Authors:** Abdullateef Bako, Saeed K. Ojolowo

**Affiliations:** 1 Department of Urban and Regional Planning, Faculty of Environmental Sciences, University of Ilorin, Kwara State, Nigeria; 2 Department of Urban and Regional Planning, Faculty of Environmental Design and Management, University of Ibadan, Oyo State, Nigeria

**Keywords:** flood, preparedness, spatial knowledge, Victoria Island, Lagos, Nigeria

## Abstract

There is inadequate flood preparedness in Victoria Island, Lagos, Nigeria; because when the flood struck on 08 July 2017, several properties were destroyed without any extant means to salvage them. This article investigated the relationship between spatial knowledge and flood preparedness in Victoria Island. The variables employed to measure spatial knowledge include knowledge of: elevation of land, distance between Lagos lagoon and Atlantic Ocean, characteristics of surrounding water bodies and building–plot ratio. Major roads were used to subdivide Victoria Island into four zones. Zone A had 799 buildings, zone B had 813 buildings, zone C had 749 buildings and zone D had 887 buildings. Of the total 3248 buildings, 344 buildings were selected, and one household head per building was selected and systematically sampled. A multinomial logit regression model was used in data analysis at *p* ≤ 0.05. The findings revealed that spatial knowledge accounted for only 25.8% of the explanation of inadequate flood preparedness. Only 6.1% of the respondents could distinguish height from elevation; those who explained density and setbacks correctly were 7.85% and 12.2%, respectively. Respondents who stated the distance between Lagos lagoon and Atlantic Ocean correctly and exhibited means of preparedness were 13.7%, respectively. However, 74.4% described the primary and secondary water bodies and their flow pattern correctly. Although inadequate spatial knowledge did not statistically account for poor flood preparedness, yet majority of the respondents neither prepared adequately for the annual flood event, nor exhibited adequate spatial knowledge. Therefore, other factors require investigation, whilst residents should acquire spatial flood-related education to influence their sense of flood preparedness.

## Introduction

The International Federation of Red Cross and Red Crescent Societies (IFRC) reported the number of disasters that struck across the world from 2004 to 2013, and it was found that the frequency of flooding was highest during this period. Flooding was reported 443 out of 686 times in Africa, resulting in hydro-meteorological disasters, 343 out of 846 times in America, 702 out of 1328 times in Asia and 49 out of 121 times in Oceania. Globally, in 2004, a total of 117 569 out of 176 615 people affected by hydro-meteorological tragedies were victims of flooding. In 2007 and 2010, the estimated number of people who were victims of flooding included 177 840 out of 212 875 people and 188 870 out of 333 527 people, respectively; whilst, in 2013, 32 051 out of 92 679 people were victims of flooding (IFRC 2014).

The occurrence of flooding and their impact on human beings are similar in Nigeria. Flooding in various parts of Nigeria has displaced millions of people, destroyed properties, disrupted socio-economic activities, contaminated water resources and facilitated the spread of water-borne diseases (Ojolowo 2016). Over 28 (80%) of the 36 states in Nigeria were devastated by flood in July 2012 (Wahab 2013). Some of the states that were severely affected include Kebbi, Kogi, Anambra, Plateau, Oyo, Bayelsa and Lagos. The impact of the 2012 flooding was very high in terms of human, material and production losses, with 363 people being killed, 5851 injured, 3 891 314 affected and 387 153 displaced (Federal Government of Nigeria – FGN 2013). These flooding events have been attributed to change in climatic variables (Douglas et al. 2009), excessive rainfall (Agbola et al. 2012), urban development in floodplains (Adelekan 2010; Wahab & Ojolowo 2017, 2018; Zheng & Qi 2011) and indiscriminate municipal solid waste disposal in water channels (Ojolowo & Wahab 2017). The value of destroyed physical and durable assets caused by the 2012 flooding in the most affected states of Nigeria has been estimated to be N1.48 trillion or its equivalent of $9.5 billion (FGN 2013).

Floods are the most common and recurring disaster in Lagos State, particularly in the littoral communities. Flood has become an annual event in Victoria Island and its environs since 2012. These coastal communities are vulnerable to flooding, owing to their proximity to the Ocean (Ojolowo 2016), location in low-lying areas (Adelekan 2012), population growth and irregular urban development (Wahab & Ojolowo 2017), wetland loss (Taiwo 2009) and sea-level rise (World Bank 2006). Urban development in coastal areas requires adequate spatial knowledge, particularly in this era of climate change, which will guide development to avert flooding and its associated calamities. Spatial knowledge, according to Kuipers (1978), is the:

[*K*]nowledge about the physical environment that is acquired and used, generally without concentrated effort, to find and follow routes from one place to another, and to store and use the relative positions of places. (pp. 129–153)

In this context, it is the knowledge of the physical environment that informs decision-making in location of buildings in disaster-free areas and in the development of land with eco-friendly techniques.

Disaster preparedness should go beyond readiness measures that expedite emergency response, rehabilitation and recovery, which results in rapid, timely and targeted assistance. It is also achieved through community-based approaches and activities that build the abilities of people and communities to cope with and minimise the effects of a disaster on their lives. Takao et al. (2004) opined that flood experience and flood risk perception are functions of flood preparedness. According to Oikawa and Katada (1999), flood experience drives personal awareness and the perceived risk of floods. Oliver (1975) had observed that previous flood experience and the duration of that experience increase the residents’ awareness of future hazards and forces them to take special measures against floods. Blaikie et al. (1994) found that people make their decisions on the assumption of a particular risk of which they have some experience; thus, all strategies for coping with disasters have a perceived precedent (Takao et al. 2004). It is commonly suggested that people are naturally very concerned about flood disasters and that flood experience affects a person’s preparedness for floods. However, Haque (1997) suggested that preparedness is not always associated with flood experience and its anticipation.

Conversance with space characteristics (height of land above sea level [ASL], closeness to water bodies, soil type, size of plot, percentage of developable plot, and setbacks from accessing roads and airspace between buildings) in terms of requirements for development of all types (residential, industrial, commercial, recreational, institutional and transportation) is necessary to avert climate change-related disasters, particularly flooding. Other forms of knowledge, such as traditional (Rai & Khawas 2019; UNISDR 2008), technological (Deken 2007) and biblical (Coppola 2011), have been investigated to unravel how the harbingers of such knowledge have been able to prepare for floods and subsequently reduced exposure to risks. However, knowledge of space characteristics and the ability to reduce exposure to flood hazards amongst residents of flood-prone areas have not been given much attention.

**FIGURE 1:**
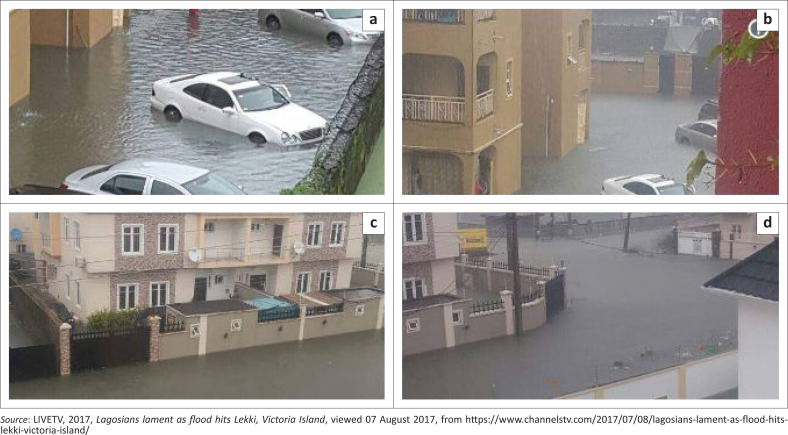
Some flooded buildings in July 2017 at Victoria Island.

Residents inadvertently occupy buildings sited in flood-prone areas and fail to prepare for inundation probably because of poor spatial knowledge. The majority of the residents of Victoria Island were caught unaware during the July 2017 flooding (Figure 1). This exposed them to the risks of floods. Therefore, this study investigated the knowledge of spatial phenomena amongst residents of Victoria Island and the subsequent preparation for flooding, in order to reinvigorate spatial knowledge in disaster management.

## Conceptual underpinning

### Knowledge

The understanding of the logical sequence of how a phenomenon operates provides insights into developing an antidote to either curb its manifestation or contain it. The knowledge of the characteristics of a hazard guides people to develop strategies against its calamities. Knowledge plays significant roles in making decisions against disastrous events. It is the only hint required to prepare for eventualities. Knowledge refers to beliefs, attitudes and expertise that people hold in memory about a topic (Hallahan 2001). It is also defined as a belief that is true and justified (Darwin 2003). The belief cannot be justified unless there is an event witnessed by the custodian of the knowledge that informed the belief. According to Robinson (1971), knowledge is not an event; rather, it has events closely connected with it, notably its origin, that is coming to know or learning, and its ending, which is being forgotten or otherwise ceasing, and its recalls or realisations whenever we bring to mind or remember that we know. These events related to knowledge are referred to using different words, including *learn, recall, realise, apprehend, see, perceive, observe, recognise, understand* and *come to know* (Robinson 1971). Knowledge empowers the bearer in managing phenomena and guides in making decisions to lessen the harsh impact of an impending disaster, if it is impossible to stop. Environmental knowledge (climate change, land-use changes and natural resource degradation) acquired through man–land interactions over the years had revealed the contributions of climate change, land-use changes and natural resource degradation to the incidence of hydro-meteorological and non-hydro-meteorological disasters. Recommendations for carbon emission reduction (Howarth 2014), tree planting and sustainable resource use (Sahney, Benton & Falcon-Lang 2010) are all knowledge-based responses to mitigate the impacts of disasters on man. However, man continues to suffer from environmental disasters on a daily basis across the globe. The efficacy of indigenous knowledge amongst people in reducing disaster risks has been demonstrated on the Island of Simeulue, Aceh, Indonesia during 2004 Tsunami, when only seven casualties were recorded out of 83 000 vulnerable population (Rahman, Sakurai & Munadi 2017; UNISDR 2005, 2015). Indigenous knowledge refers to communities’ knowledge practices – formed over numerous generations – arising from their ability to deal with and understand their environment in particular contexts and places (Rahman et al. 2017). It can potentially be disrupted through the breakdown of traditional, oral communication channels; the movement of communities to another place on a daily basis and a lack of interest in learning indigenous knowledge (Granier 1998). As a result, there is a need to look at other solutions that can serve as a guide to vulnerable residents.

### Preparedness

Preparedness signifies the knowledge and abilities acquired by people and communities to effectively forestall, respond to and recover from the impacts of likely, imminent or current hazard events or conditions (ISDR 2009). It is a defensive strategy that encompasses all actions taken prior to a disaster event targeted at lessening the disruption of life-support services, loss of life and property, damage to infrastructure and the environment, which enables governments, communities and individuals to rapidly prepare and respond efficiently to disaster-related alerts (Government of India-United Nations Development Programme (GOI/UNDP) 2002). Disaster preparedness refers to a combination of short- and long-term approaches that minimise the negative effects of natural disasters, prevent their impacts on assets, and escape certain peak values or their consequences. Deductively, disaster preparedness is more economical and humane, and showcases the level of intelligence about impending disaster well beyond emergency response, which encapsulates reactions during the crisis to salvage lives and properties (UNISDR 2005). It is usually based on command-and-control and short-term response strategies.

Preparedness embodies *knowing, understanding, recognising* and *apprehending* the interrelationships and behaviour of variables that stimulate spatial phenomena, inform the nature of the strategies likely to be adopted to curb the occurrence, reduce the impacts or completely vacate its path (relocate) (UNISDR 2015). This cannot be separated from other components of disaster management, as they are interrelated. It includes all processes of coming up with plans that have clear goals and objectives, with specific roles and responsibilities for the players, including government, non-governmental organisations, corporate bodies, community-based organisations, civil society organisation, organised labour and private individuals (philanthropists). It is a fundamental step, which ensures that during a disaster, the response is effective, timely and appropriate. The preparedness plan should include identification of causes, evacuation procedures and routes, command and communication procedures, and locations of emergency shelters. This includes training of vulnerable people on how to react to disasters peculiar to their areas and personnel saddled with the responsibility to rescue during the disaster, as in the case of Island of Simeulue, Aceh, Indonesia during 2004 Tsunami (UNISDR 2005).

## Victoria Island, Lagos, Nigeria

Victoria Island is one of the communities in Eti-Osa local government area (LGA) of Lagos, Nigeria (Figure 1). It is geographically situated along latitudes 6°23′N and 6°41′N and longitudes 2°42′E and 3°42′E. It is bounded in the north by Ikoyi, the east by Lekki, the west by Apapa and the south by Atlantic Ocean. It enjoys a daily temperature of about 27 °C because of the influence of the two air masses – the maritime south-west monsoon winds and the continental north-east dry winds from the continental interior.

Victoria Island is one of the affluent neighbourhoods in Lagos. Real estate investment in Victoria Island alone is worth over $12 bn (Lagos State Government 2017). However, exposing lives and properties to floods in Victoria Island shows lack of seriousness to protecting the valuables by all stakeholders.

**FIGURE 2:**
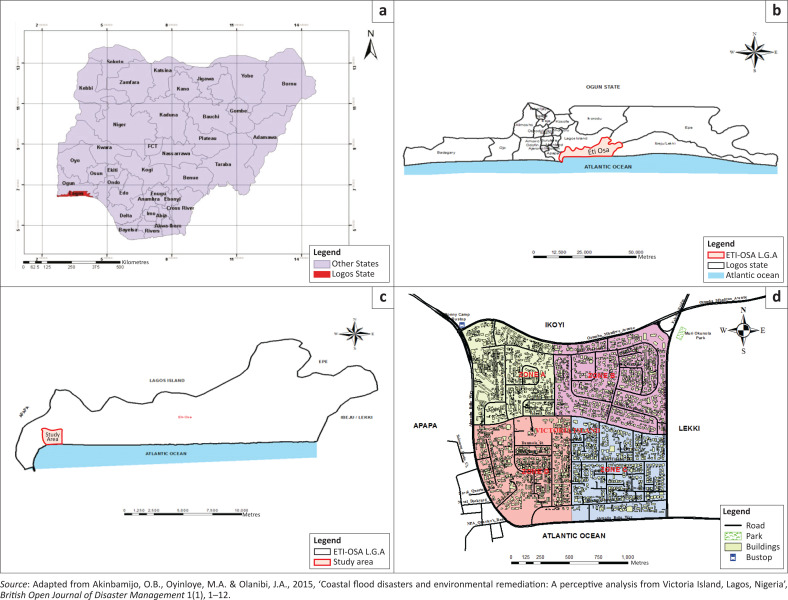
Victoria Island in Eti-Osa LGA Lagos, Nigeria.

On 08 July 2017, the areas devastated by floods include Ajah, Ikoyi, Lekki and Victoria Island; others were Lekki Phase II, Osapa London, Victoria Garden City, Ikoyi, Banana Island, Badore, Bugije, Igbo Efon and Awoyaya (Figure 1). The Lagos State Government attributed it to high tide of the lagoon, which slowed down the flow of rainwater from drainage channels (Iruoma 2017). The information attested to why flooding is more rampant and disastrous in Victoria Island. The area usually witnesses combined vigour of rainfall and ocean surge annually. Many business outfits and residential buildings are subjected to risks of flooding. Furthermore, Victoria Island is highly vulnerable to floods because it is bounded in the south and north by Atlantic Ocean and Lagos Lagoon, respectively, with the highest point not more than 10 metres (m) ASL (Figure 2).

## Materials and methods

The information used to measure spatial knowledge amongst respondents included knowledge of the elevation of land, knowledge of the distance between Lagos lagoon and Atlantic Ocean, knowledge of characteristics of surrounding water bodies and knowledge of the building–plot ratio. The extent of Victoria Island and the approximate distance between Lagos lagoon and Atlantic Ocean were delineated and calculated on Quantum Geographical Information System (QGIS), respectively. The digital elevation model data obtained from the website of United States Geological Survey (USGS) were analysed with ArcGIS 10.3 to produce the Triangular Irregular Network, which is used to analyse the elevation of land. Global Positioning Systems (GPS) was used to obtain the geographical coordinates of the sampled buildings. The collected geographical coordinates were converted to kmz file in ArcGIS 10.3 environment and imported into Google Earth for identification and subsequent measurement of plot and the percentage developed. However, those who have completely paved their compound were identified during the field survey. The statutory developable areas of the plot were obtained from the Lagos State Building Control Agency.

In order to determine the sampling frame, the study area was divided into four zones demarcated by major roads. Zone A had 799 buildings, zone B had 813, zone C had 749 and zone D had 887 buildings (Figure 2 and Table 1) adapted from the study of Akinbamijo, Oyinloye and Olanibi (2015). The sample size was determined at 5 confidence and 95% interval levels to obtain 344 out of 3248 buildings. Three-hundred and forty-four is 10.59% of 3248; therefore, 10.59% of buildings and household heads for each zone were calculated (Table 1) and systematically selected. The starting point was randomly selected using the table of random numbers. In order to determine the sampling interval, the number to be sampled was divided by the number of houses in each zone. The household heads were asked to define elevation, state the distance between Lagos lagoon and Atlantic Ocean and namecheck surrounding water bodies. Other items on the questionnaire include knowledge of size and percentage of developable plot, density, occupation, length of residence, reasons for living in Victoria Island and flood preparedness strategies. In order to test their knowledge of surrounding water bodies in Victoria Island, an unlabelled map showing all the water bodies was presented to the respondents to name.

**TABLE 1:** Sampling procedure.

Zone	Area (hectares)	Number of buildings	Sample size†
A	55	799	85
B	73	813	86
C	65	749	79
D	68	887	94

**Total**	**261**	**3248**	**344**

† , 10.59% of buildings and subsequent household heads.

The data obtained were analysed with both descriptive (tables, charts and percentages) and inferential (logistic regression) statistical models using the Statistical Packages for Social Scientists (SPSS), International Business Machines (IBM) version 20. Frequencies and percentages were used to present the data. The descriptive statistics enable the presentation of frequencies and percentages for better understanding of the variables, whilst logistic regression, also called a logit model, is used to analyse whether the respondents have knowledge of spatial phenomena (dichotomous outcome variables) and to determine the contributions of each variable to flood preparedness. In the logit model, the log odds of the outcomes (flood preparedness) are modelled as a linear combination of the predictor variables (knowledge of elevation of land, knowledge of the distance between Lagos lagoon and Atlantic Ocean, knowledge of characteristics of surrounding water bodies and knowledge of the building–plot ratio).

**FIGURE 3:**
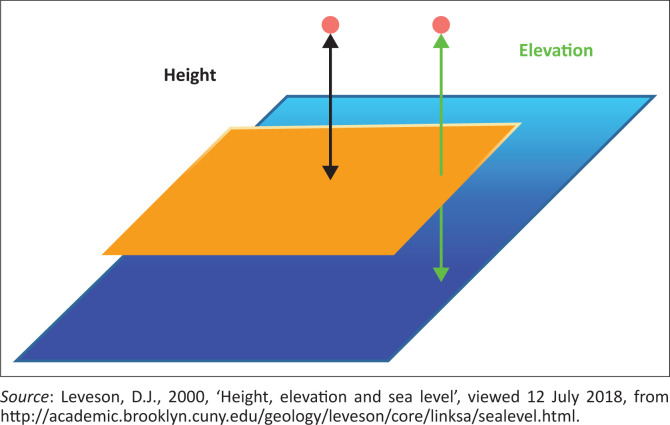
Height, elevation and mean sea level.

During the fieldwork, after enquiry the respondents were made aware of the fact that elevation was the height of a point above (or below) sea level, height as the vertical distance of a point to a horizontal surface, and sea level or mean sea level as the average height of the surface of the sea. In addition, Figure 3 is presented to each respondent to enable them understand the level of his or her exposure to floods in order to make adequate preparedness when the seasons approach. They were also informed that elevation is obtained by observing and measuring the rise and fall of tide on an open coast hourly over a 19-year period to determine the mean height of the sea. This information was provided in order to enable respondents to improve their knowledge of environment.

### Ethical consideration

This article followed all ethical standards for a research without direct contact with human or animal subjects.

## Results and discussion

### Spatial knowledge and flood preparedness

A logistic regression analysis was performed to ascertain the relationships between spatial knowledge (elevation of land ASL, distance between Lagos lagoon and Atlantic Ocean, characteristics of surrounding water bodies and building–plot ratio) and likelihood of flood preparedness. The primary data collected were transformed into binary data (adequate spatial knowledge = 1 or inadequate spatial knowledge = 0).

The analysis revealed the estimated Cox and Snell’s *R*^2^, which generally imitates multiple *R*^2^ based on ‘likelihood’ (Table 2). The Cox and Snell’s *R*^2^ of 0.258 suggested that the four independent variables in the logistic model accounted for only 25.8% of the explanation why residents of Victoria Island failed to prepare for floods of 08 July 2017. Nagelkerke’s *R*^2^ was estimated to be 0.469, indicating a good, but not strong relationship between spatial knowledge and flood preparedness.

**TABLE 2:** Binary logistic regression results showing likely effect of spatial knowledge on flood preparedness.

Step	−2 log likelihood	Cox and Snell *R*^2^	Nagelkerke *R*^2^
1	171.714	0.258	0.469

Note: Estimation terminated at iteration number seven because parameter estimates changed by < 0.001.

Inadequate preparedness exposed residents of Victoria Island to floods as shown in Table 3, which implies that 68.1% of the responses were correctly classified for flood preparedness and 94.9% for otherwise. Overall, 91.3% were correctly classified. This is a considerable improvement on the 86.3% (Table 4) correct classification with the constant model and shows that the model with predictors is better, which revealed that majority of the respondents were exposed to flooding because of inadequate preparedness.

**TABLE 3:** Binary logistic regression results for classification and prediction of flood preparedness.

Observed	Predicted		
	Preparedness		Percentage correct
	No preparedness	Prepared	
**Step 1: Preparedness**			
No preparedness	282	15	94.9
Prepare	15	32	68.1
Overall percentage	-	-	91.3

Note: The cut-off value is 0.500.

**TABLE 4:** Block 0: Binary logistic regression results for spatial knowledge and flood preparedness.

Observed	Predicted		
	Preparedness		Percentage correct
	No preparedness	Prepared	
**Step 0: Preparedness**			
No preparedness	297	0	100.0
Prepare	47	0	0.0
Overall percentage	-	-	86.3

Note: Constant is included in the model. The cut-off value is 0.500.

Table 5 shows that out of all the variables applied to measure spatial knowledge in the logistics, only knowledge of the distance between Lagos lagoon and Atlantic Ocean (*B* = 3.253, *p* = 0.000) contributed significantly to reasons why residents prepared inadequately for floods. It means that every one-unit increment in understanding the exposure to flooding because of the distance between ‘Lagos lagoon and Atlantic Ocean’ amongst residents will increase the likelihood of preparing for flooding by 3.25 times. Others, such as knowledge of elevation of land (*B* = −0.916, *p* = 0.441), knowledge of characteristics of surrounding water bodies (*B* = 1.832, *p* = 0.079) and knowledge of the building–plot ratio (*B* = 0.990, *p* = 0.406), were not. The regression function is −4.466 – 0.916’x^1^ + 3.253’x^2^ + 1.832’x^3^ + 0.990’x^4^. These results corroborated the findings where only 13.7% of respondents showed evidence of knowledge of the distance between Lagos lagoon and Atlantic Ocean and preparation for flooding.

**TABLE 5:** Binary logistic regression for effect of variables in the equation for spatial knowledge on flood preparedness.

Step 1†	B	s.e.	Wald	df	Sig.	Exp(B)	95% CI for EXP(B)	
							Lower	Upper
Knowledge of elevation of land	−0.916	1.189	0.594	1	0.441	0.400	0.039	4.115
Knowledge of the distance between Lagos lagoon and Atlantic Ocean	3.253	0.544	35.709	1	0.000	25.867	8.900	75.183
Knowledge of characteristics of surrounding water bodies	1.832	1.043	3.085	1	0.079	6.246	0.809	48.250
Knowledge of building–plot ratio	0.990	1.192	0.691	1	0.406	2.692	0.261	27.821
Constant	−4.466	1.006	19.718	1	0.000	0.011	-	-

B, beta; s.e., standard error; df, Degree of Freedom; Sig., significance level; Exp(B), exponential beta; C.I., confidence interval.

† , Variable(s) entered on step 1: Knowledge of elevation of land, knowledge of the distance between Lagos lagoon and Atlantic Ocean, knowledge of characteristics of surrounding water bodies and knowledge of the building–plot ratio.

### Elevation of land

**FIGURE 4:**
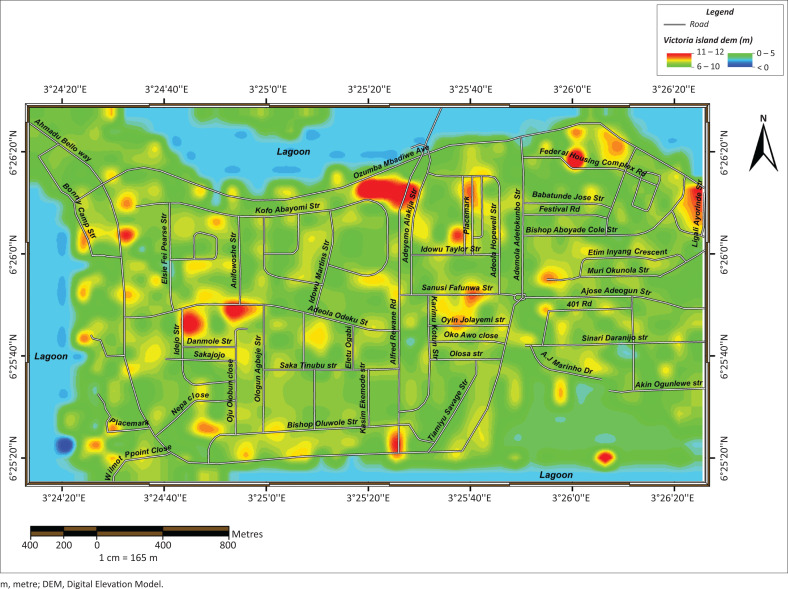
Elevation analysis of Victoria Island.

Elevation is the height of points above (or below) sea level (ASL). The analysis of elevation of Victoria Island is presented in Figure 4. The results revealed that the majority of the areas close to Atlantic Ocean and Lagos Lagoon are below the mean sea level in Victoria Island. These points are found around Ahmadu Bello Way by Bishop Oluwole Street, Nigerian Television Authority (NTA), Legico, and Bonny Camp; Adeola Odeku by Idejo Street and areas around Lagoon Restaurant. As revealed by the analysis, the majority of the areas in Victoria Island are situated on land that is ≥ 5 m ASL. These areas where the elevation falls between 6 m and 10 m are Adeyemo Alakija by Ozumba Mbadiwe Avenue, Federal Housing Complex, Ligali Ayorinde, Idowu Taylor and Oyin Jolayemi. Others included Afred Rewane Road by Atlantic Ocean, Anifowoshe by Adeola Odeku and Idejo by Danmola Street. The highest point ASL recorded is 12 m. These points dotted the landscape of Victoria Island, as shown in Figure 4. They are around Eko Close (around Ozumba Mbadwe/Kofo Abayomi Streets), Akin Adesola/Ahmadu Bello Way, Adeola Odeku/Goriola Streets and Adeola Odeku/Idejo Streets. Others included Kofo Abayomi/ Apese Streets, National Open University areas (Around Legico) and a portion within Bonny Camp. Based on the knowledge of elevation amongst the residents, only 6.1% were able to conceptually distinguish height from elevation. The majority (63.7%) of the residents gesticulated to describe elevation, whilst 30.2% were indifferent.

### Distance between Lagos lagoon and the Atlantic Ocean

**FIGURE 5:**
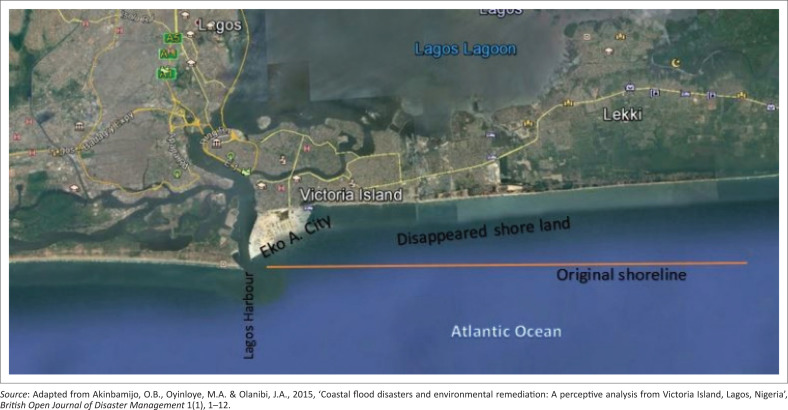
Relational location of Victoria Island, Lagos.

The distance of buildings to principal water bodies in Victoria Island is another significant factor worthy of investigation. As shown in Figure 5, Victoria Island is situated between Lagos Lagoon and the Atlantic Ocean (Akinbamijo et al. 2015). The two important water bodies are approximately 1.66 kilometres (km) (1660 m) apart. Victoria Island is enveloped by Lagos Lagoon in the north and the Atlantic Ocean in the south. These geographical traits influence accessibility both locally and internationally, as well as moderate ambient temperature, and make the Island more habitable. This is evident from the calibre of structures and the class of people who occupied the Island. The low elevation of ≥ 12 m ASL with the global sea-level rise occasioned by global climate change makes the island more susceptible to inundation. Although there had been intermittent flash flooding on the Island, it has become an annual event since 2012 (Ojolowo & Wahab 2017).

With regard to the knowledge of the respondents on the distance between Lagos lagoon and Atlantic Ocean, only 47 (13.7%) of them suggested that the distance is about 1500 m – 1600 m. Of these, 10.6% were geographers, 25.5% were land surveyors, 19.1% were town planners, 12.8% were civil engineers, 14.9% were estate surveyors, 17.0% were mechanical engineers and 10.6% were accountants. Apart from the accountants, all other professionals have to do with spatial measurements. Therefore, it is expected that they are able to correctly quantify the distance, as it has much to do with their safety during the rainy seasons. However, they were unable to state categorically the distance in either metres or kilometres. This shows that the residents who are professionals and were supposed to enlighten others fall short of expectations. Most (297 [86.3%]) of the respondents descriptively described the distance; 27.3% explained it as long, 32.3% as short and 26.7% as very short. Of these respondents, 4.4% were broadcasters, 6.4% retirees and 7.1% were contractors. Others included 11.4% bankers, 17.8% businesswomen and 52.9% businessmen. The types of occupation revealed that the island is occupied by affluent and educated people, who are expected to have the intellectual ability to understand spatial traits that could expose residents to floods, the reverse is the case in this study. Therefore, their inability to adequately prepare for floods exposed their poor knowledge of space.

### Surrounding water bodies

Apart from Atlantic Ocean and Lagos Lagoon that are prominent in Victoria Island, other water bodies pass through to empty their waters into Atlantic Ocean. The knowledge of these water bodies is essential to prepare adequately for movement out of the Island during natural phenomena, such as flooding. Five Cowrie creek extend from Banana Island to Bonny Camp and flows into the Lagos harbour- or Commodore channel to empty water from Lagos Lagoon into Atlantic Ocean. The Itirin canal spreads from the Kuramo waters in Victoria Island, runs through the premises of Mobil Producing Nigeria head office and flows into Five Cowrie creek. It is a significant primary drainage canal that helps to remove rainwater from most parts of Victoria Island during rainfall. Another canal passes through Ligali Ayorinde and flows into the Atlantic Ocean.

One-quarter (25.6%) of the respondents could not identify the Five Cowrie creek, Itirin or Ligali Ayorinde canal. This showed that they did not make enough enquiry about the environment of the Island before moving in and on moving in. By inference, they were indifferent to environmental traits that could harm them in their immediate environment. Despite this, the majority (42.0%) of the respondents have been living on the island for close to 9 years; they did not bother to acquaint themselves with their environment. For others, 9.1% moved into the island before 10 years and 25.0% before 7 years, whilst 23.9% moved in before 6 years.

The remaining 74.4% of the respondents were able to provide vivid descriptions of the primary and secondary water bodies on the island and their flow patterns, perhaps, because of their longer stay on the island. About 5.0% of them had lived on the island for 32 years, 11.1% lived for 29 years, 63.2% lived for 20 years and 20.7% for 19 years. These respondents were connected to their residences by either ownership or family affinity. Of the 74.4% respondents who satisfactorily described the water bodies, 29.3% owned the buildings and had lived there for 20–32 years. About 60.7% who had lived there for 19–20 years, indicated that their parents owned the buildings. It is expected that they would understand the drainage pattern of the island and make adequate preparation for flood management, because it has become an annual event in the last 5 years, as indicated by 87.9% of the respondents.

### Size and percentage of developable plot

Victoria Island is a low-density residential area, with plot size ranging between 1500 m^2^ and 2500 m^2^ statutorily. More than half (57.9%) of the sampled buildings were built on 1550 m^2^ plots, 21.3% on 1200 m^2^, 13.7% on 1800 m^2^, 4.1% on 2300 m^2^ and 3.0% on 2500 m^2^. Density is not only a ratio of the number of people inhabiting a given piece of land to its area but also a measure of the intensity of development of land. Density standard is fixed in order to ensure that the available land is used economically and to an optimum extent in order to make sure that development considered ecological conditions of the proposed environment.

According to Vagale (1970), three to four detached bungalows are developable on 0.4 hectare (ha) (1 acre) to accommodate 30–35 persons in a low-net density residential area; 65–80 persons are expected to inhabit 8–10 semi-detached and row housing; whilst 120–140 persons live in 14–16 multiple-family dwellings (see Table 6 for characteristics of medium-density and high-density residential development). Only 7.85% of the respondents had knowledge of density and were able to conceptualise it, which included 1.74% of civil engineers, 2.62% of town planners and 3.49% of land surveyors. The majority (92.15%) of them could neither conceptualise density nor relate it to the building–plot ratio.

**TABLE 6:** Recommended densities for residential developments.

Type of dwellings	Net density	
	No. of dwellings per 0.4 ha	No. of persons per 0.4 ha
**Bungalow (detached)**		
1. Low density	3–4	30–35
2. Medium density	4–6	35–50
3. High density	6–8	50–65
**Semi-detached and row housing**		
1. Low density	8–10	65–80
2. Medium density	10–12	8–90
3. High density	12–14	90–120
**Multiple-family dwellings**		
1. Low density	14–16	120–140
2. Medium density	16–18	140–160
3. High density	18–20	160–180

*Source*: Vagale, L.R., 1970, ‘Development control: Its importance in regulating urban growth’, Course Paper No. 4, The Polytechnic, Ibadan.

ha, hectare.

Regulations on building coverage in Section 17, sub-section 1–5 of the Lagos State Physical Planning and Development Regulations of 2005 stipulate maximum of 50% land development for low-density residential areas, such as Vitoria Island. This is to encourage percolation of rain and used water, to discourage surface run-off causing inundation and soil erosion (Wahab & Ojolowo 2017). A minimum of 20% of a plot is specified for landscaping in low-density residential areas. In order to further control the density of physical development, the number of permissible dwelling units is laid down for different land users in Lagos State and spelt out in Section 19 (1–4). Sub-section 1 stipulates a maximum of 10 units per hectare in Victoria Island (Wahab & Ojolowo 2017), which is higher than what Vagale (1970) stipulates. However, the majority of the sampled buildings violated the statutory building–plot ratio stipulated for Victoria Island.

Of all the buildings sampled, only 14.8% have not been redeveloped. Out of 14.8% (51), 80.4% had erected chalets on the space originally left for landscaping and let it out. About 31.7% of buildings had four chalets, 29.3% of buildings had three chalets, whilst 39.0% of buildings had one chalet. The space left had been paved completely, disallowing infiltration of storm water. The remaining 85.2% had redeveloped the plot into multiple-family storey buildings (see Figure 1); both the density and building–plot ratio regulations were violated. About 37.9% of buildings were newly constructed two-storey buildings; 33.1% of buildings were three-storey buildings; 21.8% of buildings were four-storey buildings, whilst 7.2% of buildings were five-storey buildings. The redevelopments commenced in 2003, with 6.5% of the reconstructed buildings, 7.8% rebuilt in 2005, whilst 19.1% were rebuilt in 2006. About 23.5% of buildings were rebuilt in 2007; those rebuilt in 2008 were the highest (35.5%); and the least (7.5%) were rebuilt in 2010.

Apart from developing beyond the statutory building–plot ratio, the remaining space left as setbacks was completely concretised. Sixty-eight percent of the respondents revealed that they knew that the setbacks of residential buildings at the right, left and rear airspace is usually 3 m. However, 12.2% of the respondents indicated 3 m as the minimum distance, and that it could be larger depending on the intensity of development and the available land. The remaining 19.8% could not categorically state the distance, but were aware that there should be space.

### Spatial knowledge and preparedness strategies

Generally, only 6.1% conceptualised elevation, 13.7% absolutely stated the distance between their residences and Atlantic Ocean, whilst 7.85% demonstrated knowledge of the building–plot ratio. However, 74.4% described the characteristics of surrounding water bodies. Because of the poor knowledge, the majority of residents were caught unaware on 08 July 2017 when the waters from Atlantic Ocean flooded Victoria Island. All residents were trapped (Figure 1), except an unknown man who was reported on one of the social media platforms paddling a canoe along Ahmadu Bello Way probably to recreate or escape from the flooded community. Definitely, he had bought the canoe in anticipation of the flood. This is the power of knowledge; he had acquired necessary information about the community before he moved in.

The study revealed that the majority of residents of Victoria Island did not have sufficient spatial knowledge that could influence their decision to take precautionary measures before the incidence of flooding. Relocation to another building outside or within Victoria Island was the only strategy identified during this study. Only 13.7% revealed that they relocated to their own buildings on the Mainland on the first day of the flood. A total of 58.1% residents moved to the upper floors; out of this, 47.9% relocated upstairs within the same buildings, whilst 52.1% did so at other buildings where their family members were residing in Victoria Island. The remaining 28.2% of them relocated to the residences of their family members on the Mainland (Table 7).

**TABLE 7:** Preparedness and response to flood.

Preparedness and response	Frequency	Percentage
Relocate to own building on Mainland	47	13.7
Relocated upstairs	200	58.1
Moved to residences of their family members on Mainland	97	28.2

**Total**	**344**	**100.0**

Respondents who relocated to the Mainland (13.7%) to their own building on the first day of the flood were those who had prepared before the incidence of the flood, as they indicated that the apartments on the Mainland have been reserved for occasional use and contingencies. The relocation of others (86.3%) was spontaneous and shows non-preparedness. Their relocation to other apartments within Victoria Island, despite their socio-economic status and repeated flooding since 2012, revealed inadequate spatial knowledge of and subsequent poor preparedness strategy for flood management. None of the respondents had sufficient food items for flood management. Furthermore, none of the buildings studied had any known preparedness strategy that could reduce the impact of flooding on the occupiers. Hence, all residents living on the ground floor lost one household material or the other to the flood. It was expected that every resident would have devised means of salvaging his or her property before the incidence of floods; however, because of the poor spatial knowledge all of them were exposed to the risks of floods. The respondents did not have any preparedness strategy in place, which could reduce the impact of floods on their lives and properties; hence, they were at the mercy of nature during the period of flooding.

## Conclusion and recommendations

Preparation for eventuality, logically, is borne out of the knowledge of either the natural or the artificial forces behind the incident. The residents of Victoria Island were caught unaware on 08 July 2017 because of inadequate spatial knowledge; despite the fact that flooding had been occurring annually since 2012, only 13.7% of the respondents exhibited the act of preparedness. Living rooms were flooded, electronics and other household materials were destroyed, and expensive cars – that could have been moved out of the Island – were damaged. Poor spatial knowledge is one of the factors that expose urban residents to the risks of floods; because all the four variables used to measure spatial knowledge in the logistic model accounted for only 25.8% of the explanation why residents of Victoria Island failed to prepare for floods of 08 July 2017. Therefore, there is a need to search for other solutions or reasons for inadequate flood preparedness in Victoria Island.

The majority of the people neither know the distance between Lagos Lagoon and Atlantic Ocean nor know how farther their residences were from Lagos Lagoon or Atlantic Ocean. They neither explained the drainage pattern nor understood the elevation characteristics of the Island. The output of the combined forces of the association that exist amongst plot–building ratio, nearness to water bodies, drainage pattern and elevation was not known to the majority of residents of Victoria Island.

The exposure to catastrophic events results from inadequate knowledge of the factors that influence their occurrences. Knowledge is indispensable in the successful management of any phenomenon that is potentially very dangerous, be it natural or anthropogenic. Therefore, Lagos State Ministry of Physical Planning and Urban Development (LSMPPUD) should enlighten the residents and developers on the forces behind flood in order to enable them to make adequate preparation. This could be carried out by identifying community associations through which every member would be reached. In order to achieve efficient preparation for the annual flood events, the LSMPPUD should enact a building code that will compel building owners on Victoria Island to redevelop their buildings to multi-floor and make the ground floors free of permanent structures, so that each time there is flood, there would be enough space for the floodwater to flow unhindered. This will not only facilitate infiltration of storm water but also encourage home owners to landscape compound for outdoor activities and discourage total pavement of compound that usually leads to flow of storm water and subsequent inundation.
